# Synthetic Cements

**Published:** 1913-08-15

**Authors:** George C. Evans


### SYNTHETIC CEMENTS.

BY GEORGE C. EVANS, D.D.S.

In view of the pros and cons on the subject of this cement as expressed in the last issue of this journal a few important remarks on the subject may be in order. In regard to successes and failures there is no cement that has ever been introduced that requires so much care, exactness and skill in its mixing and introduction into the cavity as does Synthetic Cement. The manufacturers state distinctly that the cement has to be mixed or spatulated in two (2) minutes and the introduction into the cavity performed and entirely completed in another two (2) minutes. Now is this always within this time? I think not. Again it has to be properly spatulated and mixed to just the right consistency respecting liquid and powder. Now is this also done? I think I can safely say that if the first mentioned part is the latter is not.

I am not taking the position that we have "it" at last. Not by any means, but in Synthetic Cement I have found a wonderful acquisition to my resources in that direction. Tendency not to maintain its shade and darken in time is that which I am most distrusful of. Nevertheless, I have this cement in labial cavities in the front teeth alongside of porcelain fillings in cases going on about two years, and I can only perceive a slight change. For this reason I am at present inclined to use a shade a trifle lighter than it should be to match. At times when a cavity has to be instantly and easily filled for a patient troublesome to manage, it is a very convenient agent to have on hand. If in a few years the cement will require renewal, this can also be comparatively easily done.

A few points in regard to its use. Having the cavity prepared and carefully protected from moisture (rubber dam is preferable) wipe out the cavity with alcohol and dry with warm air. Have every instrument you are going to use, or likely to use, out and in line on your bracket. Have your glass slab dry, clean and cold. Place your liquid in drops to the right and your powder to the left. The powder should be in excess of what is needed as you have no time to take from bottle. With a absolutely clean bone spatula or one of those agate expensive ones that are rather clumsy and break—as mine have when I dropped them,—bring over some of the powder and incorporate it in such a portion of the liquid as will seem to be sufficient to form the required pellet. Spatulate rapidly, then add more powder. Try and use the required amount of liquid at once, so as not to have to add to it. Continue to add powder until you have it in a thin doughy consistency. In spatulating the cement sticks annoyingly to the spatula but quickly rake it off on the edge of the slab and again and again rub and turn with the spatula until the cement takes on that semi-translucent appearance characteristic of a true mix. All the time keep your eye on the minute hand of your clock or watch to see you do not extend beyond the two minutes; it is even better to make it a few seconds short of that time if you can, as it gives you that much extra time to introduce it into the cavity, but the spatulation must nevertheless be thorough.

Before commencing to mix, wash your hands and especially the fingers with soap and pumice so as to have them absolutely free from all discoloring matter. Then with white vaseline from a tube smear the fingers and rub the vaseline well over them and wipe with a soft linen towel so that to all appearance and touch the vaseline is removed. On the back of the index finger of the left hand smear it with an imperceptible amount of vaseline smoothly laid on.

The question with me in the beginning was should I knead this cement just before I introduced it into the cavity or place it there from the spatula. I have used it both ways. I get the best results by transferring it from the spatula to between the points of the fingers of my left hand and rapidly kneading it till just that consistency is reached which facilitates its introduction into the cavity. In its introduction a small quantity should be first pressed in the deep angular parts with a small instrument, then the main portion in one unbroken pellet, if possible, inserted and the whole uniformly compressed. In proximal cavities I use a strip of thin platinum 38 gauge. I place vaseline on the surface of the platinum and then wipe it off so that the quantity applied will not be enough to come off on the cement. This will permit the platinum to glide over the cement and compress and even gloss the surface. The instruments you use to work on any part of the surface of the cement are liable to stick to it unless vaseline is used as on the platimum. An inperceptible quantity can be quickly applied to the instrument by rubbing it on the back of the surface of the index finger, where an also imperceptible quantity only has been applied.

The insertion of the cement must be accomplished in its entirety inside of four (4) minutes or less from the time the mixing of the cement was commenced.

In a few minutes the cement and the adjoining surface of the tooth is to be entirely covered with sticky wax which I allow to remain until the next day. If the above rules are followed, Synthetic Cement gives the results I have been obtaining from it. *Dental Scrap Book.*

